# Evaluation of Dental Students’ Skills Acquisition in Endodontics Using a 3D Printed Tooth Model

**DOI:** 10.14744/eej.2021.07088

**Published:** 2021-11-22

**Authors:** Ove PETERS, Raymond SCOTT, Ana ARIAS, Ella LIM, Frank PAQUÉ, Sam ALMASSI, Samer HEJLAWY

**Affiliations:** 1. Department of Endodontics, University of the Pacific, Arthur A. Dugoni School of Dentistry, San Francisco, CA, USA; University of Queensland, School of Dentistry, Brisbane, Qld, Australia; 2. Department of Endodontics, University of the Pacific, Arthur A. Dugoni School of Dentistry, San Francisco, CA, USA; 3. Department of Endodontics, University of the Pacific, Arthur A. Dugoni School of Dentistry, San Francisco, CA, USA; Department of Conservative Dentistry, Complutense University, Madrid, Spain; 4. Department of Preventive Dentistry, Periodontology and Cariology, School of Dentistry, University of Zurich Dental School, Zurich, Switzerland

**Keywords:** 3D printing, education, root canal obturation

## Abstract

**Objective:**

The purpose of this prospective quantitative study was to assess the improvement of skills among pre-clinical dental students who practiced root canal obturation on a 3D-printed tooth model.

**Methods:**

Preclinical students at the dental school (n=145) enrolled in the 2-week endodontic rotation course were invited to participate in the study. Four alphabetically distributed intact groups of first-year students were randomly allocated to either the control or the experimental group that obturated canals of a 3D-printed tooth. The plastic model was obtained from a microCT scan and based on an STL data set. The model was an identical replica of a natural mandibular molar that had been instrumented, ready for obturation. The control group did not obturate the tooth model but received identical instruction. Later in the course all students obturated an extracted human mandibular molar tooth. Technical obturation quality was assessed by two blinded evaluators. Radiographs were used to evaluate obturation length and density. Inter-observer reliability of average performance scores was calculated with the intra-class correlation coefficient for both consistency and absolute agreement. Obturation skills of those who practiced with the model were statistically compared to students who did not use the model with the Mann-Whitney U-test.

**Results:**

Inter-observer reliability was very high for both consistency and absolute agreement. No significant differences were found in obturation skills between the experimental and control group (P>0.05).

**Conclusion:**

Under the condition of this study, dental students’ obturation skills did not significantly improve by further practicing obturation using a 3D-printed model.

## Introduction

The rationale of obturation during a root canal treatment is to establish a fluid-tight barrier from the orifice of the canal to the apical foramen that protects the periapical tissues from ingress by microorganisms (1). Moreover, it has been shown that obturation quality impacts the outcome of root canal treatment (2). Treatment outcome may specifically be affected by the extension of root canal filling material. Short root fillings, more than 2mm short of radiographic apex, and long root fillings, extending beyond the radiographic apex, resulted in poorer outcomes (3, 4). A lower incidence of apical periodontitis has also been related to the absence of voids in root canal fillings (5). Conversely, improved outcomes were found for root canal treatments that extended within 2 mm of the radiographic apex and were free of voids (6).

**Highlights**

• The limited availability of natural teeth is a challenge in endodontic courses.
• There is an increase in utilization of 3D printed teeth that requires validation.
• Acquisition of clinically applicable skills needs to be verified.
• In this study, there was no significant improvement of obturation skills through the use of 3D printed teeth.

The success of initial root canal treatment performed by skilled clinicians has been reported to be as high as 90% (3, 6). The success rates of treatments provided by novice dental students or inexperienced dental practitioners is noticeably lower (7). Notably, a high percentage of inadequate root canal obturation quality has been reported among dental students (8), in spite of an extensive standardised pre-clinical training (9).

Therefore, dental educators continuously look for ways to optimise teaching methods to improve the skill level of students in preclinical endodontics (2). Currently, there is a trend for pre-clinical dental education to utilise both extracted teeth and plastic tooth replicas (10). Extracted teeth as training models have a number of limitations, among them difficulty finding suitable teeth, non-standardized training, difficulty in evaluating students’ performance, and the possibility of cross infection (11). However, natural teeth provide a better understanding of real anatomy and provide the tactile sensation of human enamel and dentine (12, 13). Plastic teeth and 3D printed teeth pose no risks for infection, may be fabricated in large numbers and their standardization allow better assessment of students’ performance (9). Initially, plastic teeth used for training were based on artificially designed models or drawings of average human teeth and did not reproduce complex root canal anatomy. More recently, rapid prototyping made it possible to print virtually unlimited numbers of identical copies of a natural tooth from CBCTs and high-resolution computed tomography (microCT) scans of natural teeth (10, 14). This way, exact external and internal morphologies of natural teeth can be replicated (15). Such tooth replicas manufactured from accurate scans of natural teeth enjoy a better acceptance among educators than conventional plastic models. However, one group of students performed poorer using microCT-based replicas because of the more complex anatomy of these models and consequently rated them poorer (16). The 3D printed replicas are still limited in their ability to fully mimic the hardness or radiopacity of dentine due to the resin that is used for 3D printing (10).

The aim of this prospective quantitative study was to assess if superior obturation skills were acquired via the obturation of 3D printed tooth replicas by dental students. The null hypothesis was that there would be no difference between the control and experimental groups in root canal obturation skills.

## Materials and Methods

The design of this study was approved by the University’s Institutional Review Board (IRB Protocol # 17-90). The ability to enrol with informed consent was offered to all first-year dental students when giving information about the study and requesting their volunteer participation. All students who chose to participate in this study gave their written consent.

### Participants and design

The study was performed as part of the regularly scheduled four consecutive 2-week endodontic block courses at the School of Dentistry. This course consists of standard lectures followed by hands-on exercises on extracted natural teeth.

All 145 students at the School of Dentistry who participated in the 2-week pre-clinical endodontic course were invited to participate in this study. At the beginning of the academic year, students had been equally distributed into four groups using non-random, alphabetical categorization for all the preclinical instruction. The four intact groups with 36 or 37 students per group were randomly allocated to either the experimental or the control group. All students received the same lecture on obturation with the method of lateral compaction. The control and experimental groups obturated the same number of canals (n=6).

**Figure 1.**
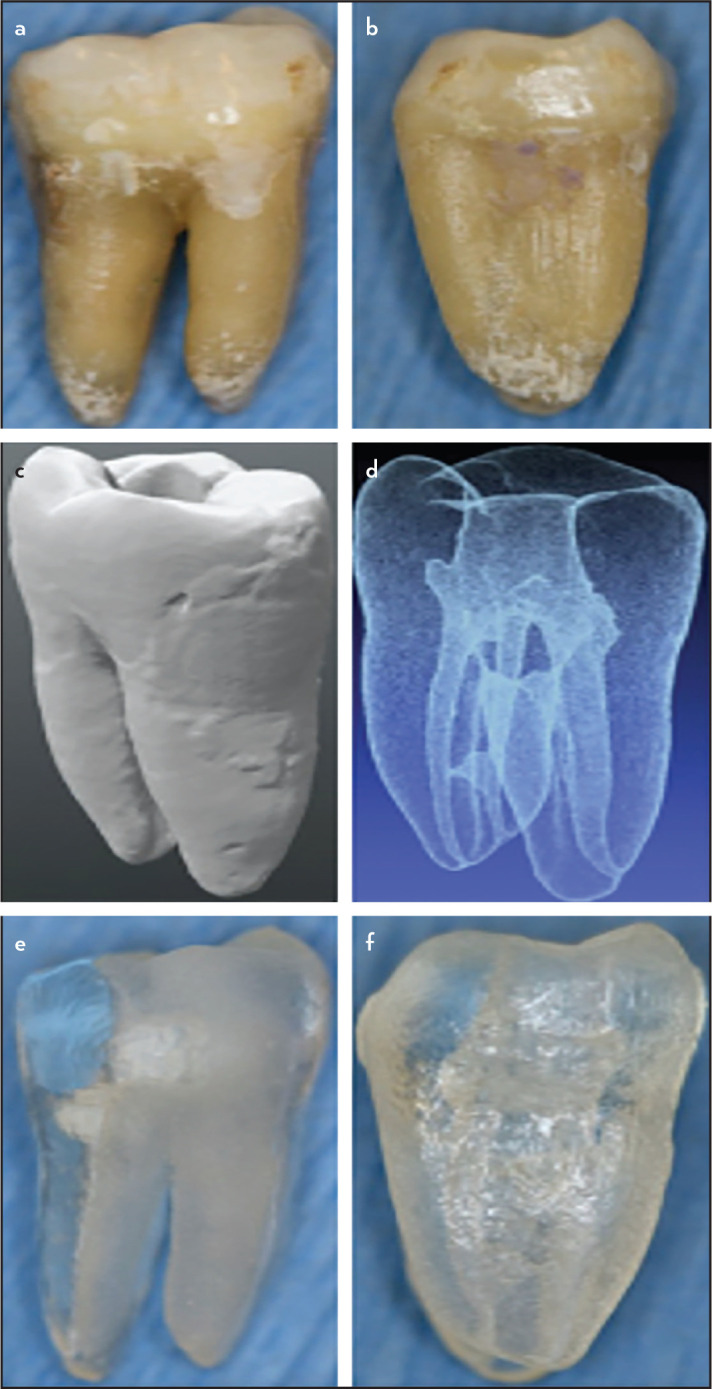
Construction of the tooth model, buccal (a) and mesial (b) view of the extracted tooth, external (c) internal (d) .stl image, buccal (e) and mesial (f) view of the 3D-printed plastic tooth model

Students in the experimental group were further given a presentation describing the 3D printed plastic tooth model that included viewing a virtual 3D model of the tooth. A microCT scan (isotropic resolution 20 μm) had been previously obtained from an extracted human mandibular first molar; this tooth was accessed and shaped to working length with Vortex Blue.06 taper rotary files (Dentsply-Sirona, York, PA) to apical sizes #30, #35 and #40, respectively for mesio-buccal, mesio-lingual and distal root canals (Fig. 1a, b). Data files in STL format (Fig. 1c, d) were generated. A commercial vendor (Acadental, Overland Park KS, USA) printed the 3D tooth models for the study (Fig. 1 e, f) that students in the experimental group obturated. The students in the control group did not obturate the model but an extracted human molar.

### Data collection

All student performance data remained confidential during the collection period. Data were kept in a secure database in a password-protected computer. Once all data were collected, individual student identification information including name and student ID was replaced with a numeric code in order to maintain confidentiality of all study participants. Data were collected to assess acquisition skills; at the end of the pre-clinical course all students completed a simulated root canal treatment on an extracted mandibular molar tooth and obtained radiographs, which were used to evaluate their obturation skills.

Obturation quality was independently rated by two evaluators. In order to prevent any undue bias, evaluators were blinded from each other and to both the student and the intervention group. The evaluators used a standardized grading rubric used at the school for the preclinical course (Table 1). Two parameters were scored: length and density of root canal obturation. The average score of the two parameters was calculated for each tooth.

**Table 1.** Grading rubric for obturation assessment

	Poor (grade of 4-5)	Fair (grade of 6-7)	Excellent (grade of 8-10)
Length of root canal filling	• More than 2 mm short • More than 1 mm long	• More than 0.5 mm and less than 2 mm short • Over-extended but less than 1 mm long	• 0.5 mm short to flush
Density of root canal filling	• Multiple voids and canal space visible	• Some voids	• Well condensed

### Statistical analysis

Results were reported in aggregate form to maintain confidentiality. Using the Statistical Package IBM SPSS Statistics for Macintosh, Version 25.0 (IBM Corp. Armonk, NY, USA), inter-observer reliability of average performance scores was calculated with the intra-class correlation coefficient (ICC) for both consistency and absolute agreement. Data for both students’ average ratings of their performance and independent ratings for length and density were found not to be compatible with a normal distribution, and therefore were analysed with the Mann-Whitney U test, and the level of significance was set at 0.05 (P=0.05).

## Results

**Figure 2.**
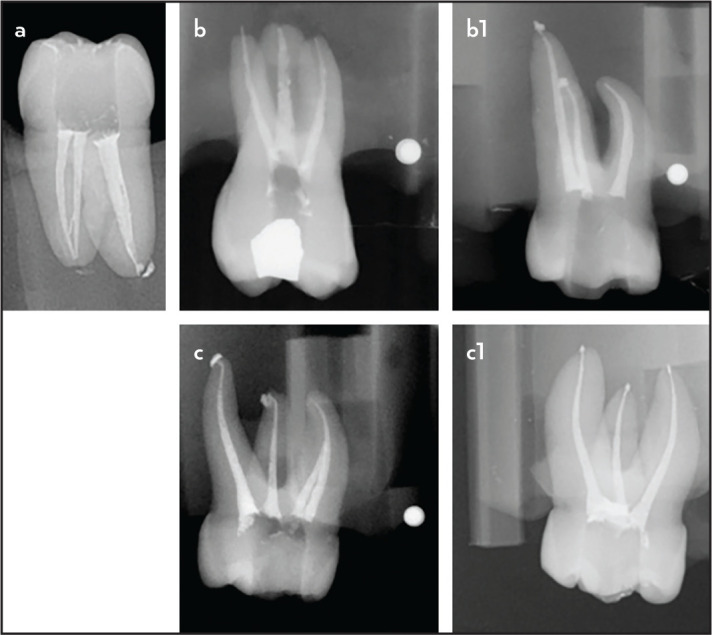
Examples of obturation outcomes of the 3D printed tooth (a) and for maxillary natural molars in groups without training (b) and after training with 3D printed teeth (c)

Valid data was obtained from 141 (97.24%) of the 145 students who signed the informed consent and voluntarily agreed to participate in the study. Inter-observer reliability was high for both consistency (ICC=0.855; Confidence interval (CI) 95% 0.803-0.894) and absolute agreement (ICC=0.847; CI 95% 0.785-0.891). Figure 2 presents examples of excellent, and similar, obturation quality in the two different groups. Mean scores and standard deviations for obturation length assessment, obturation density assessment, and the total grade for both evaluators are shown in Table 2. No significant differences were found in obturation skills between the control (mean score=13.81±2.14) and experimental groups (mean score=14.11±2.05) (P>0.05). No significant differences were found between groups independently for length or density of obturation (P>0.05).

**Table 2.** Mean scores and standard deviations for obturation length assessment, obturation density assessment and the total grade, for each evaluator separately average

	Grade	Control Group	Experimental Group
Evaluator 1	Length	7.03±1.36	7.42±1.43
	Density	6.81±1.15	6.85±1.07
	Total	13.84±2.13	14.27±2.01
Evaluator 2	Length	7.29±1.24	7.55±1.48
	Density	6.49±1.2	6.40±1.2
	Total	13.78±2.15	13.95±2.08
Means of 1 and 2	Length	7.16±1.3	7.49±1.46
	Density	6.65±1.18	6.63±1.14
	Total	13.81±2.14	14.11±2.05

## Discussion

The technical quality of root canal treatment performed by dental students and general dentists has been a subject of interest in a number of countries with differing educational systems. For instance, a study investigating the quality of root canal treatments in Taiwan reported only 30% of the teeth had received root canal treatment of adequate filling length or sealing density (17). Radiographic investigation of quality of root canal treatments in a French subpopulation demonstrated acceptable standards of treatment based on position and the density of the obturation in only 21% of the roots treated (7). A study in Jordan reported 47% of root canal obturations performed by dental students to be satisfactory in term of length, density, and taper. Considering the length alone, 61% of the roots had fillings of adequate extent in relation to the radiographic apex (18). A retrospective study conducted in Turkey (19) evaluated root canal obturation performed by dental students two years post-operatively; this was by assessing quality of obturation radiographically based upon the distance between the apical end of the root filling and the radiographic apex as well as the density of obturation. The results indicated that only 54.2% of all root fillings were of adequate length and only 53.2% of all root fillings were of adequate radiographic density (19). Collectively, these studies emphasize the need for changes in teaching methods to improve the technical quality of root canal obturation performed by dental students.

It is well-known that multi-rooted teeth are technically more challenging to obturate than single-rooted teeth due to their location in the mouth and the complexity of their root canal system. (20). In fact, it has been demonstrated that clinical training over time improved performance of the students, although students still performed poorer on obturation of teeth that were located more posteriorly in the dental arch (21). In addition, mandibular molars are one of the most difficult teeth to treat successfully (6, 10).

Most dental students state that they are not confident in undertaking molar endodontics and that they require further training before they treat molars on patients (22). Considering the scarcity of appropriate teeth for preclinical training, one possible solution to enhance students’ experiences could be the use of 3D printed tooth models for additional assignments (10). No currently available tooth model appeared to possess ideal hardness to mimic natural teeth for access and instrumentation; moreover, shaping outcomes in preclinical courses with commercial tooth models varied greatly in quality. Therefore, we decided to create an obturation model of a molar tooth with defined and specific root canal shapes.

The present study intended to assess if obturation of shaped root canals in a 3D printed molar would enhance the performance of students when compared to traditional didactics and preclinical activities in the existing curriculum. 

Three-dimensional printing is a process through which plastic or resin polymers are joined in successive and incremental planar layers until the exact replica of the object is fabricated. However, there are limitations to using 3D printed teeth for teaching purposes in dentistry and specifically in endodontics (10, 12, 16, 23). Hardness of the material is an issue during access and canal instrumentation (9-11) while heat resistance is critical for the use of thermoplastic gutta percha for obturation (14, 15). On the other hand, the use of transparent resin 3D models has been recommended for endodontic training to help identifying potential procedural errors (10).

In the present study a specific clear polymer resin was used for the printing of a 3D model, with improved hardness and radiopacity. Root canal preparation was completed prior to the microCT scan acquisition to standardize root canal shape for the acquisition of students’ root canal obturation skills, independent of their canal preparation outcomes. Students used cold lateral compaction of gutta percha for obturation.

Another important aspect in the design of this study was the use of a reliable grading rubric to compare skills acquisition between the control and the experimental group. Not only the instructional design but also assessment methods may influence the results of the intervention. Inter-observer bias is a significant limiting factor in interpretation of dental radiographs (24). In the present study, two blinded evaluators graded obturation skills of dental students. The high inter-observer reliability for both consistency and absolute agreement might be attributed to the fact that the grading rubric used in the study is the same one that the Department of Endodontics use to grade root canal obturation performed by dental students. Both raters were well trained in the use of this grading rubric and used it extensively both preclinically and in the clinic. To further avoid bias, each assessment was assigned a random number and both raters were blinded to both the student and the learning intervention (25, 26).

Effective obturation of the root canal system contributes to success of endodontic treatment (2). Both length and density of obturation are believed to play major roles in the success or failure of endodontic therapy (6). Accordingly, these aspects were included in the grading rubric used to assess the performance of students. No significant improvement was found in the obturation skills of pre-clinical dental students after obturating the tooth model. Therefore, the null hypothesis was not rejected. These results might indicate that the current curriculum includes enough preclinical practice on natural extracted teeth for students to acquire skills in root canal obturation with lateral compaction.

Tooth replicas manufactured from CBCT or microCT scans of natural teeth should be considered as an adjunct to natural teeth. Further improvements in resin materials and 3D printing hardware will result in models even more realistic training tools for better skill acquisition while avoiding the use of biological tissues. In fact, after the completion of the study Acadental released their X2 Endo tooth. It is printed using two different resins that better mimic the properties and the “feel” of enamel and dentine. These teeth are now used in our preclinical endodontic courses and also by the several licensure exams in the US. It is likely that with further improvements in printing and materials, 3D-printed teeth will eventually replace extracted teeth in preclinical education (27-29). Indeed, at the present time and perhaps accelerated by the COVID-19 pandemic and subsequent need for more simulation procedures to augment clinical teaching, some dental schools have begun to produce 3D-printed tooth models themselves (10, 30).

## Conclusion

Adding a session with obturation of a new 3D printed molar tooth model to the coursework for dental students in pre-clinical endodontic training did not significantly improve their obturation skills. Further development may increase the utilization of 3D printed models in dental education.

### Disclosures

**Acknowledgements:** The support by Acadental (Overland Park, KS, USA) is gratefully acknowledged.

**Conflict of interest:** The authors deny any conflict of interest.

**Ethics Committee Approval:** This study was approved by the University’s Institutional Review Board (Date: 20/03/2017, Number: #17-90).

**Peer-review:** Externally peer-reviewed.

**Financial Disclosure:** Material support by Acadental (Overland Park, KS, USA) is gratefully acknowledged.

**Authorship contributions:** Concept – R.S., S.A., O.P.; Design – R.S., A.A., O.P.; Supervision – R.S.; Funding - S.A., O.P.; Materials - A.A., F.P.; Data collection &/or processing – S.A., R.S., S.H., E.L.; Analysis and/or interpretation – A.A., S.A.; Literature search – S.A.; Writing – S.A., O.P.; Critical Review – R.S., A.A., O.P.
